# Pregnancy Decision‐Making Among Women With Physical Disabilities: Cross‐Sectional Survey Study

**DOI:** 10.1111/1471-0528.70135

**Published:** 2026-01-14

**Authors:** Claire Z. Kalpakjian, Susan D. Ernst, Heidi J. Haapala, Melissa L. Barber, Brittany R. Orians, Lukonde Mulenga, Shannen M. McIntosh, Julia Shah, Jodi M. Kreschmer, Rebecca Parten, Sara Rosenblum, Gina M. Jay

**Affiliations:** ^1^ Department of Physical Medicine and Rehabilitation University of Michigan Medical School Ann Arbor Michigan USA; ^2^ Department of Obstetrics and Gynaecology University of Michigan Medical School Ann Arbor Michigan USA

**Keywords:** decision‐making, health disparities, physical disability, pregnancy, survey, women's health

## Abstract

**Objective:**

Understand the pregnancy informational needs and decision‐making of women with physical disabilities.

**Design:**

Cross‐sectional.

**Setting:**

Community.

**Sample:**

114 adult American women with physical disabilities who had experience making a decision about pregnancy while disabled.

**Methods:**

Online survey.

**Main Outcome Measures:**

Pregnancy information needs and decision‐making survey.

**Results:**

Among the most important factors (very much‐quite a bit) for decision‐making were risks to the baby (72.8%) and themselves (58.7%), happiness of their partner (65.8%), and long‐term effects of pregnancy on health (58.8%). Most had the least (not at all‐a little bit) knowledge about equipment to care for an infant (57.9%), how to adapt as their body changed (54.4%), or ways to care for their infant (53.5%). Most had some to a lot of confidence (66.7%) in healthcare providers’ recommendations but were highly variable about how much their individual concerns were understood. Important information (very much‐quite a bit) included other health problems (92.5%), caring for an infant (94.2%), medications (88.4%), ambulation (78.5%), balance problems (82.6%), and spasticity (68.2%); this information was often difficult to find and unhelpful. Others were helpful when engaging in realistic discussions, being emotionally supportive, and providing financial and physical support. Healthcare providers were supportive when setting realistic expectations, providing non‐biased information, discussing risks, providing reassurance, and collaborating with other specialists.

**Conclusions:**

While pregnancy decision‐making was challenging, participants had realistic concerns, often amplified by their disability and disability‐related concerns. These results highlight areas where interventions can be developed to improve the experience of pregnancy decision‐making.

## 1 Introduction

Women with physical disabilities have a similar desire for pregnancy as their non‐disabled peers, but they can also have more ambivalence, uncertainty, and doubt [1, 2]. Factors such as the importance of having a biological child [2], length of experience with their condition [3], the effects of the disability or disease on the course of pregnancy or functioning after delivery [3, 4, 5, 6, 7], or level of functional impairment [8] can influence pregnancy decision‐making. When considering a pregnancy, women also express concern, fear, or uncertainty about the ability to care for a child [1, 6, 9], burdening a partner [10] or a child [6], passing on the disability to their child [10, 11, 12], or pregnancy itself [9]. Societal attitudes about disability and negative perceptions about disability [7, 13, 14, 15] and a lack of access to knowledgeable healthcare providers and relevant information [16] also play an important role in decision‐making. For many, pregnancy decision‐making can be a protracted process when they must balance their own health and function, the health and safety of a foetus, and the potential long‐term impact of a pregnancy [16].

In the extant literature, decision‐making about pregnancy largely focuses on decisions *during* pregnancy, such as maternity care [17] and delivery methods [18], contraception [19, 20], or terminating an unintended pregnancy [21]. In contrast, literature on the decision about pursuing a *future*, intended pregnancy among women with disabilities and chronic health conditions is relatively nascent. Studies of the experiences of pregnancy decision‐making among women with multiple sclerosis [7, 22], rheumatoid arthritis [23, 24], inflammatory bowel disease [25], or epilepsy [26] point to a complex and involved decision‐making process and a need for information and guidance that is often lacking.

Given the fact that more and more women with physical disabilities and chronic health conditions are choosing to pursue pregnancy, there is a need to understand factors involved in the decision‐making experience and their informational needs. This knowledge is critical for the development and implementation of interventions to support women and their healthcare providers during this decision‐making process. In this article, we report on the results of a pregnancy informational and decisional needs survey developed specifically for women with physical disabilities [27]. This foundational work is needed to inform intervention development, health care provider training, and clinical care guidelines.

## 2 Methods

### 2.1 Inclusion/Exclusion Criteria

Eligible participants were adult American women with physical disabilities who had experience making a decision about pregnancy while disabled. Participants had to be at least 18 years old and could reside anywhere in the United States. Physical disability was defined as a loss of function or mobility restricting the individual from one or more important life activities, irrespective of cause or age at onset. To assess the severity of disability, we used responses to three items from the Behavioural Risk Factors Surveillance System [28, 29], that assess the need for assistance with daily life activities and/or personal care; those with mild, moderate, or severe impairment for at least 6 months were eligible. We excluded those with a cognitive or psychiatric impairment that precluded the capacity to provide informed consent and participate in study activities. Women with a short‐term or temporary physical impairment (< 6 months) were not eligible.

Experience with decision‐making about pregnancy was defined in several ways that were not mutually exclusive nor restricted to a single episode or time point, given that women could experience pregnancy decision‐making multiple times in their lives. For this study, the timeframe for pregnancy decision‐making was restricted to *while disabled*. Decision‐making was characterised by having one or more pregnancies, including currently being pregnant, or having considered but deciding not to pursue pregnancy. The only exclusion criteria with respect to pregnancy decision‐making was never having considered a pregnancy at any time while disabled. See Table S1 for items used to assess pregnancy decision‐making experience eligibility.

### 2.2 Pregnancy Informational and Decisional Needs Assessment Survey

The survey used in this study has been described in detail elsewhere [27] (also see the survey form in Data S1). Briefly, the survey has six sections: the experience of making a decision, information about pregnancy and disability, things affecting a decision, knowing what is important, support for making a decision, and working with healthcare providers. Items were closed‐ and open‐ended; the latter allowed respondents to elaborate on their experience with each topic. In addition, at the end of the survey, respondents were asked to give their advice to other women with physical disabilities and health care providers about pregnancy decision‐making. This study was approved by the University of Michigan IRB Health Sciences and Behavioural Sciences (Exemption #2, U.S. Common Rule 45 CFR 46.104, reference HUM00146621).

### 2.3 Recruitment Sources, Screening, and Data Collection Procedures

Participants were recruited primarily via social media postings (e.g., Facebook groups) and encouraged to share recruitment materials with their networks. Screening was conducted via a telephone interview with a trained research associate. If the participant met all inclusion criteria and was interested in participating, they provided verbal consent. Next, an interview was conducted to collect demographic information, including the cause of disability and pregnancy history (per self‐report). This history covered the number of previous pregnancies (before and after disablement, including current pregnancy), unplanned pregnancies, loss and termination, and the use of fertility treatments. Once the interview was complete, a unique link via REDCap [30] was sent to the participant to complete the online survey form. Data collection took place between July and August 2019.

### 2.4 Analysis and Synthesis of Survey Data

We used a combination of quantitative and qualitative methods to analyse and synthesise survey data. The quantitative analysis was primarily descriptive using frequencies and means. Open‐ended responses were coded thematically using an inductive approach [31] to identify frequent, dominant, and important themes [32]. Themes were identified by a single author (CZK or JS) and reviewed by a second author (RP).

## 3 Results

### 3.1 Study Participants

Of the 169 participants screened, 114 were eligible. Of those who were screened as ineligible, 21 did not have a mild to severe physical disability for at least 6 months, and 25 did not meet inclusion criteria for pregnancy decision‐making while disabled. The remaining nine were not eligible for other reasons. Among the most frequent disabling conditions represented were musculoskeletal disorders (*n* = 19), Spina Bifida (*n* = 19), Cerebral Palsy (*n* = 16), Ehlers‐Danlos syndrome (*n* = 11), Arthrogryposis multiplex congenita (*n* = 9), and Multiple Sclerosis (*n* = 10). Less common causes of disability included autoimmune disorders (e.g., lupus, rheumatoid arthritis), Muscular Dystrophy, neurological injuries or disorders, limb loss, and Fibromyalgia. Slightly more than half of the sample (*n* = 67, 58.8%) was born with a disability. Race and Hispanic ethnicity were self‐reported as mandated by the U.S. National Institutes of Health (NIH), with categories based on NIH Policy on Reporting Race and Ethnicity data.

Relative to a broader population of Americans with disability (all types), women in this sample had substantially higher levels of college or graduate education (56.9% vs. 15.4% [33], 19.7% [34], or 26% [35]) with an overrepresentation of White women (82.5% vs. 71.9% [36] or 78.0% among those with childhood‐onset disability [37]) and underrepresentation of Black women (11.4% vs. 13.3% [36] or 18% among those with childhood onset disability [37]) and Hispanic women (7.0% vs. 11% [36] or 12% [35] but higher than 5% of those with childhood‐onset disability [37]). Fewer women in this study did not have partners (neither married nor had a significant other; 26.3% vs. 36% [38]), and slightly more were married compared to their non‐disabled peers (48.2% vs. 45% [38]). See Table 1.

**TABLE 1:** Demographics of survey participants.

Characteristics	*N* = 114
Age at disability onset (if not birth; *M*, SD; range)	26.0 (9.2); 9–46
Current age (*M*, SD; range)	35.6 (9.5); 20–75
Severity of disability	
Mild	21 (18.4%)
Moderate	52 (45.6%)
Severe	41 (36.0%)
Pregnancy decision‐making status^a^ (*N*, %)	
Ever pregnant	72 (63.2%)
Pregnant since onset of disability^b^	26 (55.3%)
Currently pregnant, actively planning, or deciding whether to get pregnant	51 (44.7%)
Already made decision never to get pregnant^c^	13 (30.9%)
Already made decision never to get pregnant again^d^	7 (9.7%)
Pregnancy history (*N*, %)^d^	
Trouble getting pregnant	30 (41.7%)
Received medical treatment for trouble getting pregnant	11 (36.7%)
Gave birth to one or more children	62 (86.1%)
Loss of pregnancy < 20 weeks	32 (44.4%)
Termination of pregnancy	9 (7.9%)
Highest education completed (*N*, %)	
High school/GED	10 (87.8%)
Associates degree	9 (7.9%)
Some college (no degree)/Other	31 (27.1%)
Bachelor's degree	33 (28.9%)
Postgraduate degree	31 (28.0%)
Marital status	
Single, never married	20 (17.5%)
Married	55 (48.2%)
Significant Other	29 (25.4%)
Divorced	10 (8.8%)
Living situation (*N*, %)	
Live alone	78 (68.0%)
Live with spouse/significant other	16 (14.0%)
Live with parents	8 (7.0%)
Live with children	8 (7.0%)
Other	4 (3.5%)
Race and Ethnicity (*N*, %)	
Asian	1 (0.9%)
American Indian or Alaska Native	1 (0.9%)
Black or African American	13 (11.4%)
Native Hawaiian or Other Pacific Islander	1 (0.9%)
White	94 (82.5%)
Hispanic or Latino	8 (7.0%)
Living in a medically underserved area (*N*, %)	13 (11.4%)
Living in a health professional shortage area (*N*, %)	
Primary care	57 (50.0%)
Dental care	38 (33.3%)
Mental healthcare	50 (43.9%)

^a^ Categories are not mutually exclusive except where noted below.

^b^ Excludes those with congenital onset (*n* = 67).

^c^ Among those with no previous pregnancy (*n* = 42).

^d^ Among those who have been previously pregnant (*n* = 72).

### 3.2 Experience of Pregnancy Decision‐Making

**FIGURE 1:**
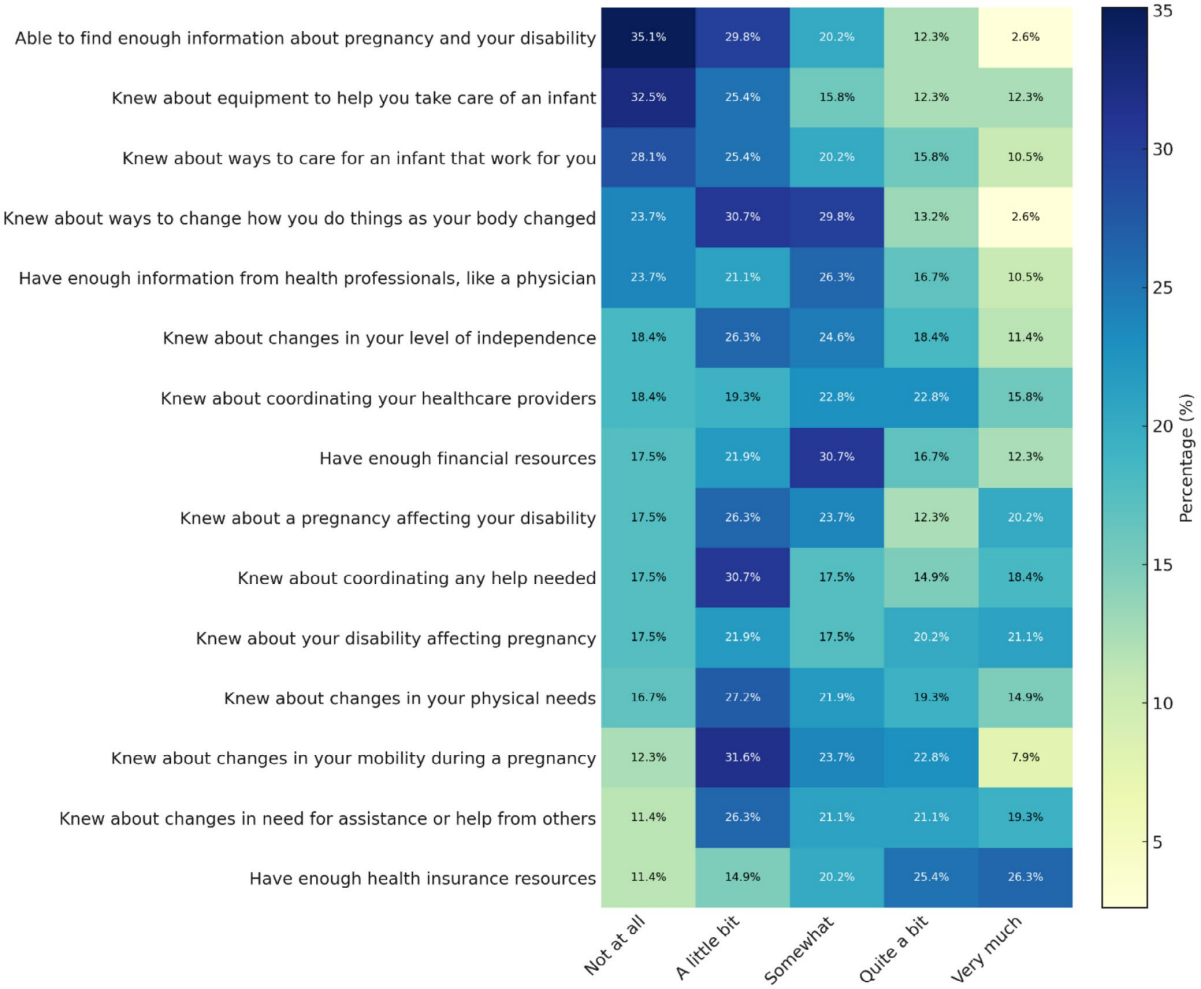
Experience of pregnancy decision‐making.

The degree of difficulty making a decision and the extent to which disability affected this was generally more difficult than easy, and was affected more by the disability than not. The overall pattern of ratings was similar in terms of disability severity (see Figures S1 and S2). Many women could not find information about pregnancy and their disability, and did not know about equipment or ways to take care of an infant that worked for them. How much they knew how they could adjust the way they carried out activities as their body changed was variable (see Figures S3 and S4). They also varied in the amount of information they received from healthcare providers and how much knowledge they had about the potential changes in their level of independence. In contrast, women generally knew about potential changes requiring assistance or help from others and had enough health insurance resources. See Figure 1.

### 3.3 Factors That Impact the Decision About Pregnancy

**FIGURE 2:**
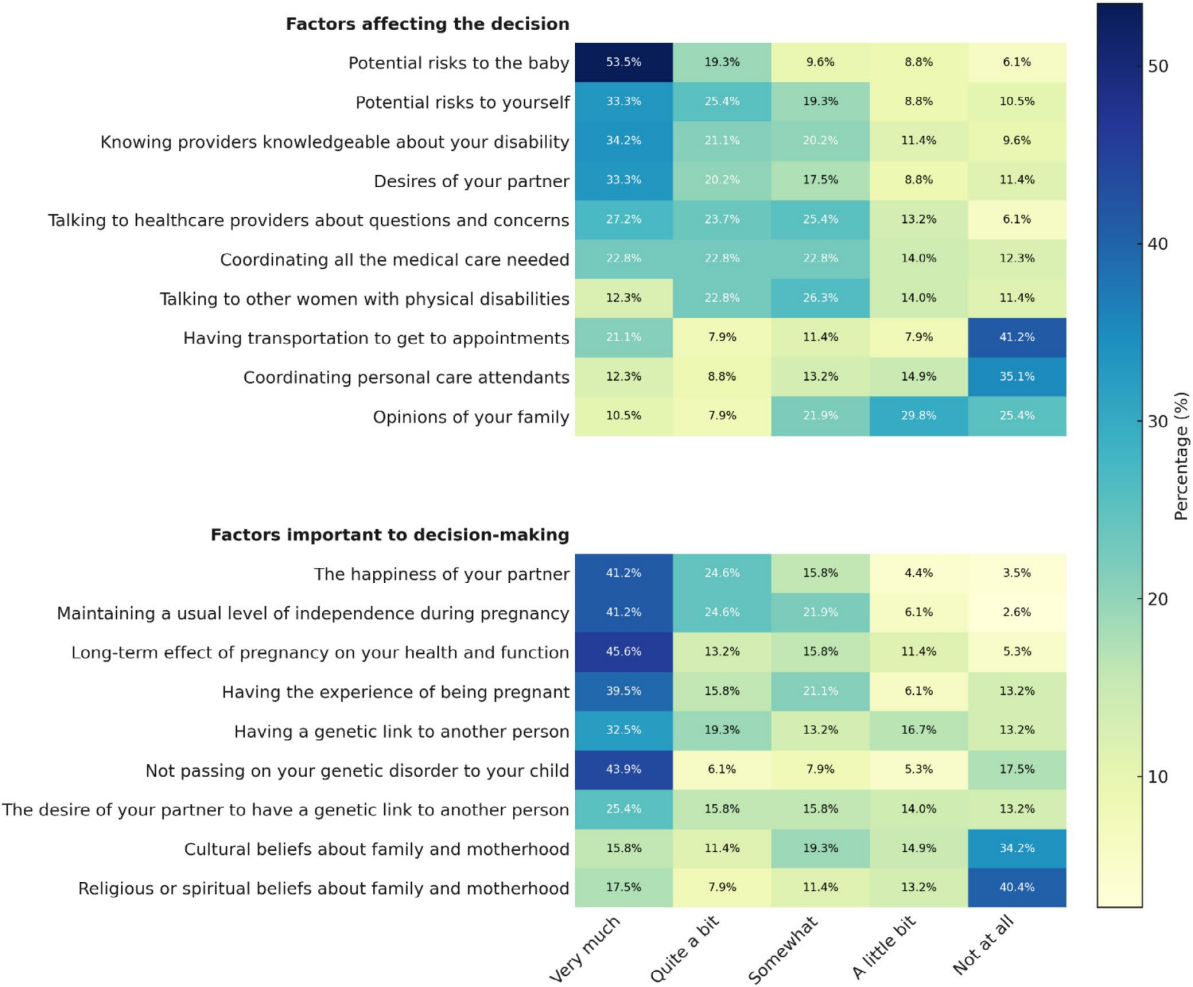
Factors affecting and important for pregnancy decision‐making.

Factors that had the greatest effect on the decision were potential risks to the baby and themselves, as well as knowing that healthcare providers were knowledgeable about their disability. Factors most important for the decision were the happiness of their partner, maintaining their usual level of independence during pregnancy, and the long‐term effects of a pregnancy on their health and function. Factors that had the least impact included having transportation to get to appointments, coordinating personal care attendants, and the opinions of family. Cultural, religious, or spiritual beliefs about family and motherhood were the least important among this sample. See Figure 2.

### 3.4 Informational Needs for Decision‐Making

The most important topics for pregnancy decision‐making were caring for an infant, medications, walking and/or ambulation, balance problems, spasticity, and other health problems. Topics such as self‐care, kidney or bladder infections, breathing difficulties, wheelchair fit, and safety of transfers were likely to be specific to certain conditions or levels of impairment and were the least endorsed in this sample. See Figure S3. “Other” topics participants added included wanting information about breastfeeding, risk of passing on their condition, financial support/insurance, safety while carrying the baby, emotional well‐being, and the effects of pregnancy on their disability/health condition. For nearly all topics, ratings of importance were considerably higher than ratings of helpfulness and ease of finding the information. See Figures S4 and S5.

The sources of pregnancy information varied depending on the topic. Healthcare providers primarily gave information about medications, but a majority of the information was also found online. In contrast, healthcare providers were a minority among sources of information about caring for an infant compared to peers and the Internet. Information about walking and/or ambulation and balance problems was evenly distributed across healthcare providers, peers, and the Internet. See Table 2.

**TABLE 2:** Sources of information about pregnancy.

Topic (*N* endorsed, % of all respondents)^a^	Source of information (*N*, % of endorsed)				
	Healthcare providers	Peers	Internet	Other	Not started looking or gotten
Caring for an infant when you have a disability (*N* = 88, 77.2%)	24 (27%)	42 (48%)	51 (58%)	10 (11%)	21 (24%)
Medications you usually take, not related to fertility or pregnancy (*N* = 78, 68.4%)	65 (83%)	13 (17%)	49 (63%)	6 (8%)	5 (6%)
Walking and/or ambulation (*N* = 78, 68.4%)	46 (59%)	25 (32%)	36 (46%)	2 (3%)	15 (19%)
Balance problems (*N* = 70, 61.4%)	35 (50%)	25 (36%)	33 (47%)	7 (10%)	14 (20%)
Spasticity or muscle tightness and cramping (*N* = 66, 57.9%)	30 (45%)	15 (23%)	29 (44%)	4 (6%)	17 (26%)
Other health problems, related or unrelated to your disability (*N* = 66, 57.9%)	46 (70%)	25 (38%)	40 (61%)	5 (8%)	11 (17%)
Self‐care, like bathing, dressing or grooming (*N* = 55, 48.2%)	18 (33%)	26 (47%)	27 (49%)	5 (9%)	11 (20%)
Kidney or bladder infections (*N* = 31, 27.2%)	20 (65%)	1 (3%)	10 (32%)	4 (13%)	7 (23%)
Breathing difficulty (*N* = 28, 24.6%)	15 (54%)	4 (14%)	12 (43%)	1 (4%)	6 (21%)
Wheelchair fit, safety and/or manoeuvring (25, 21.9%)	9 (36%)	6 (24%)	14 (56%)	4 (16%)	7 (28%)
Transfers (25, 21.9%)	10 (40%)	7 (28%)	11 (44%)	5 (20%)	4 (16%)

^a^ “Other” topics are not reported in this table.

### 3.5 Support of Others for Decision‐Making

Half or more of the participants had quite a bit/very much support from partners, friends, and families for pregnancy decision‐making. However, nearly a third reported quite a bit/very much pressure *not* to get pregnant (see Figure S6). Those most involved tended to be their partner, family, healthcare providers, and friends. The type of healthcare providers included maternal and foetal medicine physicians, physical therapists, and nurses. Other women with disabilities, including mothers with disabilities, were also mentioned. A handful of participants reported that no one else was involved in their decision‐making.

Partners, family, friends, and healthcare providers all positively impacted the decision‐making process. Specifically, they helped by engaging in realistic discussions, weighing pros and cons, and being emotionally available and supportive. Partners, family, and friends also helped with problem‐solving, such as researching different devices and adaptive equipment that could make a pregnancy easier to manage. Partners and family further supported the process by providing financial or instrumental support, helping with self and infant care, or attending doctor appointments. Partners specifically played a role in planning the timing of the pregnancy, weighing the disability impact on the pregnancy, such as having “what if” conversations, and ultimately helping them decide.

Healthcare providers were noted for their roles as counsellors by setting realistic expectations, providing non‐biased information, discussing risks, and providing reassurance. They also helped by taking the time to collaborate with other specialists. A handful of participants specifically mentioned other parents with disabilities and children of disabled parents as positively impacting their decision‐making process by sharing their own experiences with pregnancy, how they adapted, and providing reassurance. Although most had positive experiences, a minority felt that people in their lives did not support their decisions or had a negative effect on decision‐making.

Among those who did not have adequate support, many felt that they received unwanted opinions from others, most often offered by family members. For example, family could be over‐involved by questioning their decision to pursue pregnancy or suggesting termination. To a lesser extent, strangers and acquaintances, healthcare providers, and friends also contributed unwanted opinions. In terms of unmet support needs, participants most often wanted to connect with other mothers with disabilities. They also wanted more support from healthcare providers, especially more education about different risks and potential pregnancy complications, as well as more encouraging and supportive attitudes. They also wanted better access to physical and occupational therapists to help them adjust to their body's changing needs during the pregnancy and more emotional support, including speaking with a mental health counsellor about the decision.

### 3.6 Working With Healthcare Providers

While healthcare providers did not necessarily have sufficient knowledge about how pregnancy would affect the disability (and vice versa) or risks to the baby due to the disability, participants felt that healthcare providers generally understood their concerns and supported them throughout the decision‐making process. Despite some deficits in knowledge and their concerns being understood, most participants had a moderate to high degree of confidence in their recommendations (see Figure S7).

### 3.7 Advice to Other Women With Physical Disabilities and Healthcare Providers

At the end of the survey, participants were asked to share their advice for other women and healthcare providers about making a decision about a future pregnancy. Advice to other women with physical disabilities coalesced around their *agency and interdependence with others*. The importance of connecting to their peers was tied to the value of learning from others' lived experiences and the unique understanding they offer each other. This was considered an important complement to what is learned from healthcare providers and their own information gathering. Participants also commented on the importance of networks of support, needing practical help from others, and the necessity of planning. Many emphasised trusting and knowing themselves and following their heart. In this advice, there was a distinct emphasis on the importance of *self‐advocacy*, particularly in working with healthcare providers and having limited information. Relatedly, participants were keenly aware that they were planning and managing their pregnancies within a broader culture that often dismisses their desires for motherhood, requiring the strength to confront bias and discrimination in their pursuit of motherhood. For many, their advice focused on believing in themselves and wrapping the support of trusted others around them to buffer the challenges that they often faced in the decision‐making process.

Advice to healthcare providers coalesced around a theme of *being allies and partners*. This was reflected in the importance of listening and respecting a woman's knowledge of herself and her disability. Withholding judgement and providing information and advice without being overly influenced by personal opinions set the stage for good communication and trust. Women also emphasised the importance of healthcare providers reaching out to medical colleagues as much as possible to ensure comprehensive care and reducing the burden on women to find other healthcare providers to meet their needs (See themes and quotes in Table S2).

## 4 Discussion

### 4.1 Main Findings

This study builds on a growing body of qualitative studies exploring the pregnancy decision‐making experience of women with disabilities [16, 39, 40] by contributing complementary quantitative data using a survey tool designed specifically for women with physical disabilities [27]. In reflecting on their experiences, they had realistic concerns for their health and function and the health of their baby, sharing common concerns about pregnancy amplified by the presence of a disability and disability‐specific concerns. A lack of relevant information available compounded these concerns. While they enjoyed support from others, they still contended with unwanted opinions of others and negativity toward their decision.

### 4.2 Interpretation

The reproductive freedom, including making a decision whether or not to pursue pregnancy, of women with disabilities has been a longstanding challenge across world regions, even in high‐income countries such as the United States as suggested in the findings of this study. The Convention on Rights of Persons with Disabilities [41], adopted in 2006, explicitly recognises the historic denial of reproductive freedom through stereotypes that too often result in erroneous beliefs that people with disabilities are asexual and lack agency for their sexuality and reproduction. There are increasing calls to rectify these inequities through responsive and equitable sexual and reproductive health services [42, 43], education [43], autonomy and informed consent [41], and to challenge myths about disability and sexuality [44].

Several key findings from this study highlight issues that lay the groundwork for the next step in this line of research. In this sample, cultural, religious, and spiritual factors did not have a strong influence on decision‐making. However, these factors have varying degrees of importance in other cultures and world regions. For example, among women living in Middle Eastern countries, comfort when seeking information from healthcare providers is lower than for women living in Western countries [45]. Feeling ashamed or embarrassed can also create barriers for women seeking information about pregnancy from healthcare providers [46, 47]. Given the significant challenges that many women with physical disabilities face, particularly the cultural biases that they cannot be mothers [15], it is possible that those who do ultimately pursue pregnancy, or decide not to, have a high degree of autonomy. More research is needed to understand the personal, contextual, and cultural factors that influence information gathering to inform pregnancy decision‐making among women with physical disabilities.

Another notable finding in our study was the primacy of a partner's happiness in making a decision about pregnancy. In fact, this was rated as highly as maintaining a usual level of independence during pregnancy, and only slightly less important than the long‐term effects of pregnancy on health and function. There is some evidence that the happiness of couples and an alignment of fertility desires play an important role in shared decision‐making about pregnancy [48, 49]. Unfortunately, women with disabilities are more likely to experience reproductive coercion [48] than their peers [50], which increases the likelihood of an *unintended* pregnancy. While there is some evidence that worries about burdening partners [10] play a role in pregnancy decision‐making, more research is needed to understand the influence of partner happiness and other relationship quality factors on pregnancy decision‐making among women with physical disabilities.

Finally, women's confidence in their healthcare provider's recommendations did not always align with the extent to which their concerns were understood. While on its face, this may appear contradictory, the relationship itself is likely a stronger driver of confidence than the specifics of understanding specific concerns. There is growing evidence that healthcare providers' interpersonal competence [51, 52] and empathy [53] are key drivers of patient trust. Moreover, women with physical disabilities tend to be realistic about the extent to which non‐specialist healthcare providers will have expertise about their disability, and they highly value a trusting and collaborative relationship [16]. In this study, their advice to healthcare providers highlights the vital role they play in helping to set realistic expectations, providing unbiased information, seeking counsel from their colleagues, offering support, and withholding judgement. Given the challenges that many women with physical disabilities face, particularly in reproductive healthcare [16], these findings point to an important but understudied relationship that warrants further exploration to understand the influence of patient‐provider trust and relationship in pregnancy decision‐making.

### 4.3 Strengths and Limitations

A particular strength of this study was our focus on the pregnancy decision‐making process itself and engaging women with physical disabilities with diverse experiences. The diversity of our sample in terms of disability severity and cause also supports its generalizability. The mix of closed and open‐ended survey items enabled us to aggregate data and gain a deeper understanding of experience through narratives. Despite these strengths, there are several limitations. We engaged a predominantly White and well‐educated sample of American women. As such, the experiences of American women of colour, those living in other world regions, and those from different cultural backgrounds are not well represented. This is especially important to understand the additional influence of racial, ethnic, and cultural factors, and as such, limits the generalisability of results. Rather, these findings should be viewed as hypothesis‐generating for future exploration in other populations. The design of the survey focused on information needs on topics most relevant to disability and less so on general pregnancy information. As such, these data are limited for assessing the relative importance of more general topics vs. those specific to disability. For women who had multiple pregnancy decision‐making experiences, we did not ask them to distinguish these in responding to the survey questions. Finally, the range of years that women in this study had their pregnancies will have had some bearing on their experiences, given that the rise of the Internet and social media were not common or available in the 1990s.

## 5 Conclusions

While pregnancy decision‐making was challenging for many participants in this study, they had realistic concerns, which were often amplified by their disability and additional disability‐related concerns. What we have learned in this study of American women with physical disabilities can be explored further to understand the ways in which culture and world region intersect with disability to influence this experience. Such understanding is essential for the development of high‐quality, evidence‐based information and interventions that are tailored for different settings to support women with physical disabilities in making informed decisions about pregnancy.

## Author Contributions

The conceptualization and funding acquisition were accomplished by C.Z.K., S.D.E., and H.J.H., C.Z.K. provided project oversight and supervision. L.M., J.M.K., R.P., and S.M.M. were responsible for participant recruitment and data collection. C.Z.K. performed quantitative data analysis. Qualitative data synthesis was accomplished by C.Z.K., J.S., and R.P. All authors participated in data interpretation. C.Z.K. prepared the initial manuscript draft, and the critical review of the draft and approval of the final version for publication were given by all authors.

## Funding

This work was funded by the U.S. National Institutes of Health, *Eunice Kennedy Shriver* National Institute of Child Health and Human Development (NIH/NICHD) [grant no. R21 HD092526]. REDCap at the University of Michigan is supported by the Michigan Institute for Clinical and Health Research funded by the U.S. National Institutes of Health, National Center for Advancing Translational Sciences (NIH/NCATS) [grant no. UL1TR002240].

## Ethics Statement

The University of Michigan IRB Health Sciences and Behavioural Sciences approved this study under Exemption #2, U.S. Common Rule 45 CFR 46.104 on April 18, 2019, reference HUM00146621.

## Consent

All participants provided informed consent.

## Conflicts of Interest

The authors declare no conflicts of interest.

## Supporting information


**Data S1:** Pregnancy decision making survey.

**Table S1:** Screening questions for determining pregnancy decision‐making experience eligibility.
**Table S2:** Themes of advice to other women with physical disabilities and healthcare providers.
**Figure S1:** Degree of decision‐making difficulty.
**Figure S2:** How much disability severity affected pregnancy decision‐making.
**Figure S3:** Difficulty finding and quality of information on pregnancy decision‐making topics.
**Figure S4:** Ease of finding information about pregnancy among those endorsing the topic as relevant.
**Figure S5:** Helpfulness of information about pregnancy among those endorsing the topic as important.
**Figure S6:** Support from others for decision‐making about pregnancy.
**Figure S7:** Working with health care providers in pregnancy decision‐making.

## Data Availability

All data analysed in this study are available through the Inter‐university Consortium for Political and Social Research (ICPSR). The dataset is open access and includes accompanying documentation for reuse.
