# Supplementary material for: Pregnancy Decision‐Making Among Women With Physical Disabilities: Cross‐Sectional Survey Study
Source: BJOG. 2026 Jan 14;133(5):1016–25. doi: 10.1111/1471-0528.70135 (PMC12853368; doi:10.1111/1471-0528.70135)
Supplement: Supplementary file 1 — Data S1: Pregnancy decision making survey. [file BJO-133-1016-s002.pdf]

Pregnancy Decision Making Survey

Dear [fname], This survey is about making a decision about getting pregnant. We want to learn what women with physical disabilities thought about or are thinking about when making this decision, and what they need to know to make a decision that is right for them. Your responses will help us understand what information is needed, and what makes decision-making easier or harder. This survey should take about 15-30 minutes to complete. If you need a break, you can save your answers and come back. If you have any questions at any time, just reply to the email you received with this survey link. Thank you!

Experience of Making a Decision about Pregnancy

What is today's date? ______________________________

This first set of questions are about your overall experience of making a decision about pregnancy.

Experience of Making a Decision about Pregnancy

On a scale of 0 to 10, with 0 being 'very easy' and 10 being 'very hard', how easy or hard [custom_time] it to make a decision about trying to get pregnant?

____

On a scale of 0 to 10, with 0 being 'not at all' and 10 being 'completely', how much [custom_did1] your disability affect your decision making about pregnancy?

____

When thinking about this decision, [custom_did2] you feel that you [custom_know] enough about...

	Not at all	A little bit	Somewhat	Quite a bit	Very much	Not applicable
your disability affecting pregnancy?	○	○	○	○	○	○
pregnancy affecting your disability?	○	○	○	○	○	○
changes in your physical needs during pregnancy?	○	○	○	○	○	○
changes in your need for assistance or help from others during pregnancy?	○	○	○	○	○	○
coordinating any help needed during pregnancy?	○	○	○	○	○	○

When thinking about this decision, [custom_did2] you feel that you [custom_know] enough about...

	Not at all	A little bit	Somewhat	Quite a bit	Very much	Not applicable
coordinating your health care providers during pregnancy?	○	○	○	○	○	○
changes in your mobility during a pregnancy?	○	○	○	○	○	○
changes in your level of independence during pregnancy?	○	○	○	○	○	○
equipment to help you take care of an infant?	○	○	○	○	○	○
ways to care for an infant that work for you?	○	○	○	○	○	○

When thinking about this decision, [custom_did2] you feel that you [custom_have] enough...

	Not at all	A little bit	Somewhat	Quite a bit	Very much	Not applicable
emotional support from your partner?	○	○	○	○	○	○
emotional support from your family?	○	○	○	○	○	○
emotional support from your friends?	○	○	○	○	○	○
financial resources?	○	○	○	○	○	○
health insurance resources?	○	○	○	○	○	○

When thinking about this decision, [custom_did2] you...

	Not at all	A little bit	Somewhat	Quite a bit	Very much	Not applicable
have enough information from health professionals, like a physician?	○	○	○	○	○	○
feel pressure from others not to get pregnant?	○	○	○	○	○	○
find enough information about pregnancy and your disability?	○	○	○	○	○	○
know ways you could change how you do things as your body [custom_changes]?	○	○	○	○	○	○

Experience of Making a Decision about Pregnancy

[custom_time2] there anything else you [custom_did2] not know about when making a decision about pregnancy?

Information about Pregnancy and Disability

These next questions are about information that you [custom_need] to help inform your decision about pregnancy. First, you will check anything on the list that [custom_applies] to you and add anything not on the list by checking one or more of the "other" items at the end. Next, for each item you checked and ones you added, you will rate its importance, helpfulness, ease or difficulty getting, and where you got it.

Information about Pregnancy and Disability

Check any item on this list that [custom_applies] to you. At the end of the you can write in anything not on the list.

☐ Medications you usually take (not related to fertility or pregnancy)
☐ Kidney or bladder infections
☐ Other health problems, related or unrelated to your disability
☐ Spasticity or muscle tightness and cramping
☐ Breathing difficulty
☐ Balance problems
☐ Walking and/or ambulation
☐ Transfers
☐ Self-care, like bathing, dressing, grooming
☐ Wheelchair fit, safety and/or maneuvering
☐ Caring for an infant when you have a disability
☐ Other 1
☐ Other 2
☐ Other 3
☐ Other 4
☐ Other 5

Describe "Other 1"

____

Describe "Other 2"

____

Describe "Other 3"

____

Describe "Other 4"

____

Describe "Other 5"

____

Information about Pregnancy and Disability

1 How IMPORTANT [custom_time] it to have information about medications you usually take (not related to fertility or pregnancy)?

- ◯ Not at all
- ◯ A little bit
- ◯ Somewhat
- ◯ Quite a bit
- ◯ Very much

2 How HELPFUL [custom_time] the information about medications you usually take (not related to fertility or pregnancy)?

- ◯ Not at all
- ◯ A little bit
- ◯ Somewhat
- ◯ Quite a bit
- ◯ Very much
- ◯ Haven't started looking or gotten it yet
- ◯ Looked but couldn't find anything

3 How EASY [custom_time] it to get information about medications you usually take (not related to fertility or pregnancy)?

- ◯ Without any difficulty
- ◯ With a little difficulty
- ◯ With some difficulty
- ◯ With much difficulty
- ◯ Unable to get
- ◯ Haven't started looking or gotten it yet

4 WHERE did you get information about medications you usually take (not related to fertility or pregnancy)?

- ☐ Health care providers
- ☐ Peers
- ☐ Internet
- ☐ Other
- ☐ Haven't started looking or gotten it yet

5 Describe "other"

____

On a scale from 0 to 10, with 0 being low quality and 10 being high quality, what [custom_time] the QUALITY of the information you got about medications you usually take (not related to fertility or pregnancy)?

____

Information about Pregnancy and Disability

1 How IMPORTANT [custom_time] it to have information about kidney or bladder infections?

- ◯ Not at all
- ◯ A little bit
- ◯ Somewhat
- ◯ Quite a bit
- ◯ Very much

2 How HELPFUL [custom_time] the information about kidney or bladder infections?

- ◯ Not at all
- ◯ A little bit
- ◯ Somewhat
- ◯ Quite a bit
- ◯ Very much
- ◯ Haven't started looking or gotten it yet
- ◯ Looked but couldn't find anything

3 How EASY [custom_time] it to get information about kidney or bladder infections?

- ◯ Without any difficulty
- ◯ With a little difficulty
- ◯ With some difficulty
- ◯ With much difficulty
- ◯ Unable to get
- ◯ Haven't started looking or gotten it yet

4 WHERE did you get information about kidney or bladder infections?

- ☐ Health care providers
- ☐ Peers
- ☐ Internet
- ☐ Other
- ☐ Haven't started looking or gotten it yet

5 Describe "other"

____

On a scale from 0 to 10, with 0 being low quality and 10 being high quality, what [custom_time] the QUALITY of the information you got about kidney or bladder infections?

____

Information about Pregnancy and Disability

1 How IMPORTANT [custom_time] it to have information about other health problems, related or unrelated to your disability?

- ◯ Not at all
- ◯ A little bit
- ◯ Somewhat
- ◯ Quite a bit
- ◯ Very much

2 How HELPFUL [custom_time] the information about other health problems, related or unrelated to your disability?

- ◯ Not at all
- ◯ A little bit
- ◯ Somewhat
- ◯ Quite a bit
- ◯ Very much
- ◯ Haven't started looking or gotten it yet
- ◯ Looked but couldn't find anything

3 How EASY [custom_time] it to get information about other health problems, related or unrelated to your disability?

- ◯ Without any difficulty
- ◯ With a little difficulty
- ◯ With some difficulty
- ◯ With much difficulty
- ◯ Unable to get
- ◯ Haven't started looking or gotten it yet

4 WHERE did you get information about other health problems, related or unrelated to your disability?

- ☐ Health care providers
- ☐ Peers
- ☐ Internet
- ☐ Other
- ☐ Haven't started looking or gotten it yet

5 Describe "other"

____

On a scale from 0 to 10, with 0 being low quality and 10 being high quality, what [custom_time] the QUALITY of the information you got about other health problems, related or unrelated to your disability?

____

Information about Pregnancy and Disability

1 How IMPORTANT [custom_time] it to have information about spasticity or muscle tightness and cramping?

- ◯ Not at all
- ◯ A little bit
- ◯ Somewhat
- ◯ Quite a bit
- ◯ Very much

2 How HELPFUL [custom_time] the information about spasticity or muscle tightness and cramping?

- ◯ Not at all
- ◯ A little bit
- ◯ Somewhat
- ◯ Quite a bit
- ◯ Very much
- ◯ Haven't started looking or gotten it yet
- ◯ Looked but couldn't find anything

3 How EASY [custom_time] it to get information about spasticity or muscle tightness and cramping?

- ◯ Without any difficulty
- ◯ With a little difficulty
- ◯ With some difficulty
- ◯ With much difficulty
- ◯ Unable to get
- ◯ Haven't started looking or gotten it yet

4 WHERE did you get information about spasticity or muscle tightness and cramping?

- ☐ Health care providers
- ☐ Peers
- ☐ Internet
- ☐ Other
- ☐ Haven't started looking or gotten it yet

5 Describe "other"

____

On a scale from 0 to 10, with 0 being low quality and 10 being high quality, what [custom_time] the QUALITY of the information you got about spasticity or muscle tightness and cramping?

____

Information about Pregnancy and Disability

1 How IMPORTANT [custom_time] it to have information about breathing difficulty?

- ◯ Not at all
- ◯ A little bit
- ◯ Somewhat
- ◯ Quite a bit
- ◯ Very much

2 How HELPFUL [custom_time] the information about breathing difficulty?

- ◯ Not at all
- ◯ A little bit
- ◯ Somewhat
- ◯ Quite a bit
- ◯ Very much
- ◯ Haven't started looking or gotten it yet
- ◯ Looked but couldn't find anything

3 How EASY [custom_time] it to get information about breathing difficulty?

- ◯ Without any difficulty
- ◯ With a little difficulty
- ◯ With some difficulty
- ◯ With much difficulty
- ◯ Unable to get
- ◯ Haven't started looking or gotten it yet

4 WHERE did you get information about breathing difficulty?

- ☐ Health care providers
- ☐ Peers
- ☐ Internet
- ☐ Other
- ☐ Haven't started looking or gotten it yet

5 Describe "other"

____

On a scale from 0 to 10, with 0 being low quality and 10 being high quality, what [custom_time] the QUALITY of the information you got about breathing difficulty?

____

Information about Pregnancy and Disability

1 How IMPORTANT [custom_time] it to have information about balance problems?

◯ Not at all
◯ A little bit
◯ Somewhat
◯ Quite a bit
◯ Very much

2 How HELPFUL [custom_time] the information about balance problems?

◯ Not at all
◯ A little bit
◯ Somewhat
◯ Quite a bit
◯ Very much
◯ Haven't started looking or gotten it yet
◯ Looked but couldn't find anything

3 How EASY [custom_time] it to get information about balance problems?

◯ Without any difficulty
◯ With a little difficulty
◯ With some difficulty
◯ With much difficulty
◯ Unable to get
◯ Haven't started looking or gotten it yet

4 WHERE did you get information about balance problems?

☐ Health care providers
☐ Peers
☐ Internet
☐ Other
☐ Haven't started looking or gotten it yet

5 Describe "other"

____

On a scale from 0 to 10, with 0 being low quality and 10 being high quality, what [custom_time] the QUALITY of the information you got about balance problems?

____

Information about Pregnancy and Disability

1 How IMPORTANT [custom_time] it to have information about walking and/or ambulation?

- ◯ Not at all
- ◯ A little bit
- ◯ Somewhat
- ◯ Quite a bit
- ◯ Very much

2 How HELPFUL [custom_time] the information about walking and/or ambulation?

- ◯ Not at all
- ◯ A little bit
- ◯ Somewhat
- ◯ Quite a bit
- ◯ Very much
- ◯ Haven't started looking or gotten it yet
- ◯ Looked but couldn't find anything

3 How EASY [custom_time] it to get information about walking and/or ambulation?

- ◯ Without any difficulty
- ◯ With a little difficulty
- ◯ With some difficulty
- ◯ With much difficulty
- ◯ Unable to get
- ◯ Haven't started looking or gotten it yet

4 WHERE did you get information about walking and/or ambulation?

- ☐ Health care providers
- ☐ Peers
- ☐ Internet
- ☐ Other
- ☐ Haven't started looking or gotten it yet

5 If other, please describe

____

On a scale from 0 to 10, with 0 being low quality and 10 being high quality, what [custom_time] the QUALITY of the information you got about walking and/or ambulation?

____

Information about Pregnancy and Disability

1 How IMPORTANT [custom_time] it to have information about transfers?

- ◯ Not at all
- ◯ A little bit
- ◯ Somewhat
- ◯ Quite a bit
- ◯ Very much

2 How HELPFUL [custom_time] the information about transfers?

- ◯ Not at all
- ◯ A little bit
- ◯ Somewhat
- ◯ Quite a bit
- ◯ Very much
- ◯ Haven't started looking or gotten it yet
- ◯ Looked but couldn't find anything

3 How EASY [custom_time] it to get information about transfers?

- ◯ Without any difficulty
- ◯ With a little difficulty
- ◯ With some difficulty
- ◯ With much difficulty
- ◯ Unable to get
- ◯ Haven't started looking or gotten it yet

4 WHERE did you get information about transfers?

- ☐ Health care providers
- ☐ Peers
- ☐ Internet
- ☐ Other
- ☐ Haven't started looking or gotten it yet

5 Describe "other"

____

On a scale from 0 to 10, with 0 being low quality and 10 being high quality, what [custom_time] the QUALITY of the information you got about transfers?

____

Information about Pregnancy and Disability

1 How IMPORTANT [custom_time] it to have information about self-care, like bathing, dressing, grooming?

- ◯ Not at all
- ◯ A little bit
- ◯ Somewhat
- ◯ Quite a bit
- ◯ Very much

2 How HELPFUL [custom_time] the information about self-care, like bathing, dressing, grooming?

- ◯ Not at all
- ◯ A little bit
- ◯ Somewhat
- ◯ Quite a bit
- ◯ Very much
- ◯ Haven't started looking or gotten it yet
- ◯ Looked but couldn't find anything

3 How EASY [custom_time] it to get information about self-care, like bathing, dressing, grooming?

- ◯ Without any difficulty
- ◯ With a little difficulty
- ◯ With some difficulty
- ◯ With much difficulty
- ◯ Unable to get
- ◯ Haven't started looking or gotten it yet

4 WHERE did you get information about self-care, like bathing, dressing, grooming?

- ☐ Health care providers
- ☐ Peers
- ☐ Internet
- ☐ Other
- ☐ Haven't started looking or gotten it yet

5 Describe "other"

____

On a scale from 0 to 10, with 0 being low quality and 10 being high quality, what [custom_time] the QUALITY of the information you got about self-care, like bathing, dressing, grooming?

____

Information about Pregnancy and Disability

1 How IMPORTANT [custom_time] it to have information about wheelchair fit, safety, and/or maneuvering?

- ◯ Not at all
- ◯ A little bit
- ◯ Somewhat
- ◯ Quite a bit
- ◯ Very much

2 How HELPFUL [custom_time] the information about wheelchair fit, safety, and/or maneuvering?

- ◯ Not at all
- ◯ A little bit
- ◯ Somewhat
- ◯ Quite a bit
- ◯ Very much
- ◯ Haven't started looking or gotten it yet
- ◯ Looked but couldn't find anything

3 How EASY [custom_time] it to get information about wheelchair fit, safety, and/or maneuvering?

- ◯ Without any difficulty
- ◯ With a little difficulty
- ◯ With some difficulty
- ◯ With much difficulty
- ◯ Unable to get
- ◯ Haven't started looking or gotten it yet

4 WHERE did you get information about wheelchair fit, safety, and/or maneuvering?

- ☐ Health care providers
- ☐ Peers
- ☐ Internet
- ☐ Other
- ☐ Haven't started looking or gotten it yet

5 Describe "other"

____

On a scale from 0 to 10, with 0 being low quality and 10 being high quality, what [custom_time] the QUALITY of the information you got about wheelchair fit, safety, and/or maneuvering?

____

Information about Pregnancy and Disability

1	How IMPORTANT [custom_time] it to have information about caring for an infant when you have a disability?	◯ Not at all<br>◯ A little bit<br>◯ Somewhat<br>◯ Quite a bit<br>◯ Very much
2	How HELPFUL [custom_time] the information about caring for an infant when you have a disability?	◯ Not at all<br>◯ A little bit<br>◯ Somewhat<br>◯ Quite a bit<br>◯ Very much<br>◯ Haven't started looking or gotten it yet<br>◯ Looked but couldn't find anything
3	How EASY [custom_time] it to get information about caring for an infant when you have a disability?	◯ Without any difficulty<br>◯ With a little difficulty<br>◯ With some difficulty<br>◯ With much difficulty<br>◯ Unable to get<br>◯ Haven't started looking or gotten it yet
4	WHERE did you get information about caring for an infant when you have a disability?	☐ Health care providers<br>☐ Peers<br>☐ Internet<br>☐ Other<br>☐ Haven't started looking or gotten it yet
5	Describe "other"	__________________________________

On a scale from 0 to 10, with 0 being low quality and 10 being high quality, what [custom_time] the QUALITY of the information you got about caring for an infant when you have a disability?

____

Information about Pregnancy and Disability

1 How IMPORTANT [custom_time] it to have information about [info_other1]?

- ◯ Not at all
- ◯ A little bit
- ◯ Somewhat
- ◯ Quite a bit
- ◯ Very much

2 How HELPFUL [custom_time] it to have information about [info_other1]

- ◯ Not at all
- ◯ A little bit
- ◯ Somewhat
- ◯ Quite a bit
- ◯ Very much
- ◯ Haven't started looking or gotten it yet
- ◯ Looked but couldn't find anything

3 How EASY [custom_time] it to get information about [info_other1]

- ◯ Without any difficulty
- ◯ With a little difficulty
- ◯ With some difficulty
- ◯ With much difficulty
- ◯ Unable to get
- ◯ Haven't started looking or gotten it yet

4 WHERE did you get information about [info_other1]

- ☐ Health care providers
- ☐ Peers
- ☐ Internet
- ☐ Other
- ☐ Haven't started looking or gotten it yet

5 Describe "other"

____

On a scale from 0 to 10, with 0 being low quality and 10 being high quality, what [custom_time] the QUALITY of the information you got about [info_other1]?

____

Information about Pregnancy and Disability

1 How IMPORTANT [custom_time] it to have information about [info_other2]?

- ◯ Not at all
- ◯ A little bit
- ◯ Somewhat
- ◯ Quite a bit
- ◯ Very much

2 How HELPFUL [custom_time] it to have information about [info_other2]

- ◯ Not at all
- ◯ A little bit
- ◯ Somewhat
- ◯ Quite a bit
- ◯ Very much
- ◯ Haven't started looking or gotten it yet
- ◯ Looked but couldn't find anything

3 How EASY [custom_time] it to get information about [info_other2]

- ◯ Without any difficulty
- ◯ With a little difficulty
- ◯ With some difficulty
- ◯ With much difficulty
- ◯ Unable to get
- ◯ Haven't started looking or gotten it yet

4 WHERE did you get information about [info_other2]

- ☐ Health care providers
- ☐ Peers
- ☐ Internet
- ☐ Other
- ☐ Haven't started looking or gotten it yet

5 If other, please describe

____

On a scale from 0 to 10, with 0 being low quality and 10 being high quality, what [custom_time] the QUALITY of the information you got about [info_other2]?

____

Information about Pregnancy and Disability

1 How IMPORTANT [custom_time] it to have information about [info_other3]?

- ◯ Not at all
- ◯ A little bit
- ◯ Somewhat
- ◯ Quite a bit
- ◯ Very much

2 How HELPFUL [custom_time] it to have information about [info_other3]

- ◯ Not at all
- ◯ A little bit
- ◯ Somewhat
- ◯ Quite a bit
- ◯ Very much
- ◯ Haven't started looking or gotten it yet
- ◯ Looked but couldn't find anything

3 How EASY [custom_time] it to get information about [info_other3]

- ◯ Without any difficulty
- ◯ With a little difficulty
- ◯ With some difficulty
- ◯ With much difficulty
- ◯ Unable to get
- ◯ Haven't started looking or gotten it yet

4 WHERE did you get information about [info_other3]

- ☐ Health care providers
- ☐ Peers
- ☐ Internet
- ☐ Other
- ☐ Haven't started looking or gotten it yet

5 If other, please describe

____

On a scale from 0 to 10, with 0 being low quality and 10 being high quality, what [custom_time] the QUALITY of the information you got about [info_other3]?

____

Information about Pregnancy and Disability

1 How IMPORTANT [custom_time] it to have information about [info_other4]?

- ◯ Not at all
- ◯ A little bit
- ◯ Somewhat
- ◯ Quite a bit
- ◯ Very much

2 How HELPFUL [custom_time] it to have information about [info_other4]

- ◯ Not at all
- ◯ A little bit
- ◯ Somewhat
- ◯ Quite a bit
- ◯ Very much
- ◯ Haven't started looking or gotten it yet
- ◯ Looked but couldn't find anything

3 How EASY [custom_time] it to get information about [info_other4]

- ◯ Without any difficulty
- ◯ With a little difficulty
- ◯ With some difficulty
- ◯ With much difficulty
- ◯ Unable to get
- ◯ Haven't started looking or gotten it yet

4 WHERE did you get information about [info_other4]

- ☐ Health care providers
- ☐ Peers
- ☐ Internet
- ☐ Other
- ☐ Haven't started looking or gotten it yet

5 If other, please describe

____

On a scale from 0 to 10, with 0 being low quality and 10 being high quality, what [custom_time] the QUALITY of the information you got about [info_other4]?

____

Information about Pregnancy and Disability

1 How IMPORTANT [custom_time] it to have information about [info_other5]?

- ◯ Not at all
- ◯ A little bit
- ◯ Somewhat
- ◯ Quite a bit
- ◯ Very much

2 How HELPFUL [custom_time] it to have information about [info_other5]

- ◯ Not at all
- ◯ A little bit
- ◯ Somewhat
- ◯ Quite a bit
- ◯ Very much
- ◯ Haven't started looking or gotten it yet
- ◯ Looked but couldn't find anything

3 How EASY [custom_time] it to get information about [info_other5]

- ◯ Without any difficulty
- ◯ With a little difficulty
- ◯ With some difficulty
- ◯ With much difficulty
- ◯ Unable to get
- ◯ Haven't started looking or gotten it yet

4 WHERE did you get information about [info_other5]

- ☐ Health care providers
- ☐ Peers
- ☐ Internet
- ☐ Other
- ☐ Haven't started looking or gotten it yet

5 Describe "other"

____

On a scale from 0 to 10, with 0 being low quality and 10 being high quality, what [custom_time] the QUALITY of the information you got about [info_other5]?

____

Things Affecting a Decision about Pregnancy

These next questions are about things that can make a decision about pregnancy harder or easier or not affect it at all. For each question, "your decision making" refers to your decision specifically about pregnancy.

In general, what [custom_affecting] your decision making?

How much [custom_did2] each of the following affect your decision making?

	Not at all	A little bit	Somewhat	Quite a bit	Very much	Not applicable
Potential risks to yourself	◯	◯	◯	◯	◯	◯
Potential risks to the baby	◯	◯	◯	◯	◯	◯
Opinions of your family	◯	◯	◯	◯	◯	◯
Desires of your partner	◯	◯	◯	◯	◯	◯
Talking to other women with physical disabilities who have been pregnant before	◯	◯	◯	◯	◯	◯

How much [custom_did2] each of the following affect your decision making?

	Not at all	A little bit	Somewhat	Quite a bit	Very much	Not applicable
Talking to health care providers about questions and concerns	○	○	○	○	○	○
Coordinating all the medical care needed	○	○	○	○	○	○
Knowing of health care providers knowledgeable about your disability	○	○	○	○	○	○
Coordinating personal care attendants	○	○	○	○	○	○
Having transportation to get to appointments	○	○	○	○	○	○

Things Affecting a Decision about Pregnancy

Is there anything else that [custom_affecting] your decision making that we did not mention?

Things That Are Important When Making a Decision about Pregnancy

These next questions are about the importance of certain things when making a decision about a pregnancy.

How important [custom_time] ...

	Not at all	A little bit	Somewhat	Quite a bit	Very much	Not applicable
having the experience of being pregnant?	○	○	○	○	○	○
having a genetic link to another person or passing on your genes to a future generation?	○	○	○	○	○	○
maintaining your usual level of independence during pregnancy?	○	○	○	○	○	○
the possibility of a long term effect of pregnancy on your health and function?	○	○	○	○	○	○
not passing on your genetic disorder to your child?	○	○	○	○	○	○

How important [custom_time] ...

	Not at all	A little bit	Somewhat	Quite a bit	Very much	Not applicable
your religious or spiritual beliefs about family and motherhood?	◯	◯	◯	◯	◯	◯
your cultural beliefs about family and motherhood?	◯	◯	◯	◯	◯	◯
the happiness of your partner?	◯	◯	◯	◯	◯	◯
the desire of your partner to have a genetic link to another person?	◯	◯	◯	◯	◯	◯

Things That Are Important When Making a Decision about Pregnancy

Are there other things you think are important for making a decision about pregnancy that we didn't mention?

Communicating with Health Care Providers about Pregnancy

These next questions are about your experience talking with health care providers to get information, advice, or any other guidance for making a decision about getting pregnant.

**How much [custom_did2] you feel your health care providers...**	Not at all	A little bit	Somewhat	Quite a bit	Very much
[custom_understand] your concerns about pregnancy?	○	○	○	○	○
[custom_understand] what [custom_matters] to you when thinking about pregnancy?	○	○	○	○	○
[custom_support] you throughout the decision making process?	○	○	○	○	○
[custom_know] risks to you during pregnancy that might be due to your disability?	○	○	○	○	○
[custom_know] risks to your baby during pregnancy that might be due to your disability?	○	○	○	○	○
[custom_know] about how pregnancy might affect your mobility and/or physical functioning?	○	○	○	○	○
[custom_are] confident about the recommendations they [custom_give] you about pregnancy?	○	○	○	○	○

Communicating with Health Care Providers about Pregnancy

Is there anything else about your experience talking with health care providers about pregnancy that we did not mention?

Support for Making a Decision about Pregnancy

These next questions are about support and resources when making a decision about pregnancy. "Support from others" refers to how much people in your life, like a partner, family, friends and health care providers, [custom_give] you emotional support and practical support. Resources include things like information and advice from various sources.

Support for Making a Decision about Pregnancy

Who else [custom_time] involved in making this decision with you? This can be anyone, for example your partner, family, friends, or health care professionals. By 'involved' we mean things like giving you advice, helping you find information, listening and problem solving with you.

Briefly describe how others listed in the question above [custom_are] involved in making this decision.

Support for Making a Decision about Pregnancy

In general, how much support [custom_did2] you have from others in making this decision?

- ◯ None
- ◯ A little bit
- ◯ Some
- ◯ Quite a bit
- ◯ A lot

Support for Making a Decision about Pregnancy

[custom_time2] there anyone who was not involved in making this decision that you [custom_want] to be or anyone involved that you [custom_donot] want to be in making this decision?

Support for Making a Decision about Pregnancy

How much support did you get after you made your decision?

- ◯ None
- ◯ A little bit
- ◯ Some
- ◯ Quite a bit
- ◯ A lot
- ◯ Have not made a decision yet

Support for Making a Decision about Pregnancy

Is there any other support that you [custom_donot] have but [custom_need]? For example, informational materials, talking to other women with disabilities, or counseling from a health professional.

Advice to Other Women with Disabilities and Health Care Providers about Pregnancy

For these final questions, we are interested in what advice you would give other women with physical disabilities thinking about pregnancy and what advice you would give health care providers who work with these women.

Advice to Other Women with Disabilities and Health Care Providers about Pregnancy

What advice would you give other women with physical disabilities thinking about or actively planning a pregnancy?

Advice to Other Women with Disabilities and Health Care Providers about Pregnancy

What advice would you give health care providers about working with women with physical disabilities thinking about or actively planning a pregnancy?

Final Thoughts

Is there anything else you would like to add about your experience making a decision about pregnancy that we didn't cover in the previous questions?

May we contact you with any questions we have about your survey or to learn more about your experience? If yes, we'll contact you by email first and set up a time to talk more that works for you.

◯ Yes ◯ No

If you need to return to the survey at a later time, please click "Save & Return Later" below. If you have completed the survey, please be sure to click "Submit" before leaving the page. Thank you!
