# Supplementary material for: Pregnancy Decision‐Making Among Women With Physical Disabilities: Cross‐Sectional Survey Study
Source: BJOG. 2026 Jan 14;133(5):1016–25. doi: 10.1111/1471-0528.70135 (PMC12853368; doi:10.1111/1471-0528.70135)

**Supporting Materials**

## Table S1. Screening questions for determining pregnancy decision-making experience eligibility

Items for screening

1. **Have you been pregnant before, either planned or unplanned? (administered to all respondents)**

1 Yes

0 No

**2. Have you been pregnant, either planned or unplanned, *since the onset of your disability*? (skip if congenital onset)**

1 Yes

0 No

**3. Are you pregnant now, actively planning a pregnancy, or in the process of making a decision about whether or not to get pregnant? (administered to all respondents)**

1 Yes

0 No

**4. Have you already made a decision to never get pregnant? (administer if items 1, 2, and 3 are all “no”)**

1 Yes

0 No

**5. Have you already made a decision to never get pregnant *again*? (administer if item 1 or 2 is “yes” and item 3 is “no”)**

1 Yes

0 No

**Can you tell me more about that? (administer if items 4 or 5 are “yes”)**

Eligibility determination

- Eligible = response of “yes” to items 2 OR 3 OR 4 OR 5.
- Ineligible = response of “no” to items 2 AND 3 AND (4 OR 5).

## Table S2. Themes of Advice to Other Women with Physical Disabilities and Healthcare Providers

|  | **THEME** | **ILLUSTRATIVE QUOTE** |
| --- | --- | --- |
| **Advice to other women** | Talk to and connect with other women | - I would encourage other women with physical disabilities to talk to women who have similar disabilities and who have had pregnancies before. Peers are the best people to help problem solve and share tips, encouragement, and advice. |
|  | Have a network of support | - Make sure you have a good support system. The support system you have will be key throughout your entire pregnancy so it's really important that you have people that are a good support for you. It is also essential to have a good medical team before you start your pregnancy planning. So I would say before you get pregnant make sure you have a good medical team on board that way you know you'll have the help you need. |
|  | Educate yourself, do your research | - Keep seeking answers to questions and get as much info from as many sources as possible so you can make the best decisions - Every question matters so just ask them. |
|  | Find the right healthcare providers | - I would say try to find healthcare professionals who are willing to listen and answer questions. Find a support system to go over plans and ideas with and make sure their opinions are ones you trust. |
|  | Know your disability, trust yourself, follow your heart | - Only you can decide what is right for you... everyone around you can have information and opinions that all need to be considered but, in the end, you know what is right for you. - Make this decision for yourself. You are the best judge as to what you can and cannot handle. Listen to your body. - Take advice from every source then, follow your heart. |
|  | Think things through, plan ahead | - Make sure you pre-think everything: how will I carry my baby, how will I change them, how will I pick them up out of bed and off the floor. Make sure you have set up arrangements for help from neighbors or family members. If you fall out of your chair pregnant (after about 5 mos) you cannot get up on your own, so make sure you are prepared with a phone at all times. |
| **Advice to health care providers** | Listen to your patients | - Listen to your patient, she knows her body better than anyone else. Search far and wide for answers to her questions. Be prepared for the most difficult scenario but don't dwell on it. |
|  | Be willing to reach out to others | - Build strong networks in the health care community so you'll be able to connect women with disabilities to the right care team that knows about pregnancy AND her disability, and how they affect each other. Help coordinate any specialists and other resources because it's very difficult for a pregnancy woman with a disability to manage so many different aspects of care. |
|  | Be supportive, withhold judgment | - Be supportive of any women who wants to be a mother regardless of her disability status. Women with disabilities are just women first and the desire to be a mom is there regardless of a wheelchair or physical differences. Listen to their concerns, encourage them to educate you on their disability. Do your own research on other women with their diagnosis being pregnant. Reach out to your colleagues to ask for information if you're unaware. |

## Figure S1. Degree of decision-making difficulty


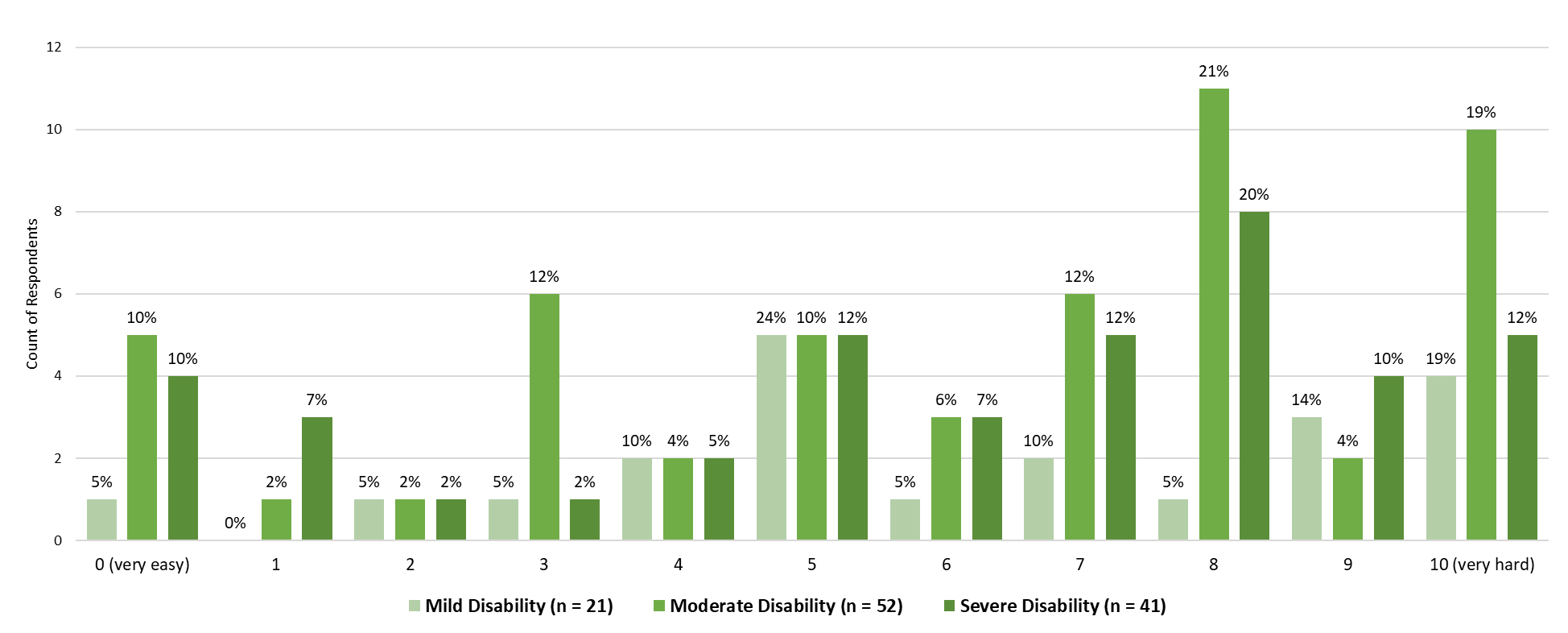


## Figure S2. How much disability severity affected pregnancy decision-making


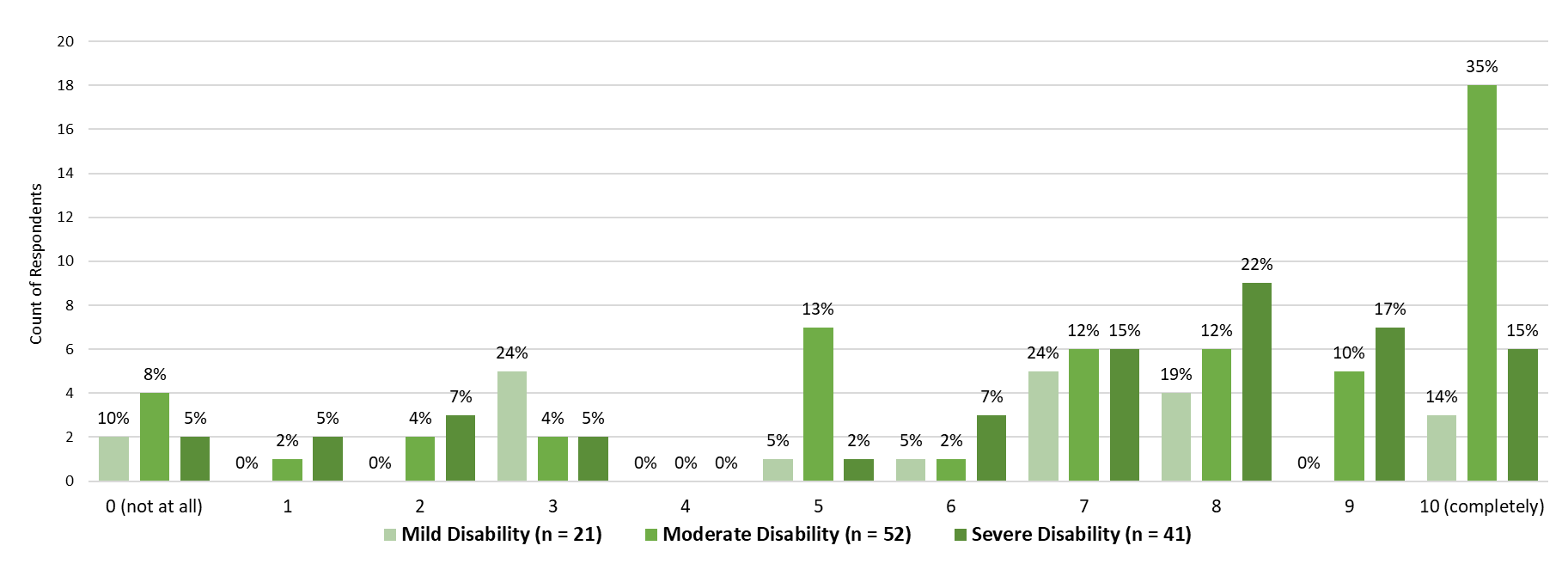


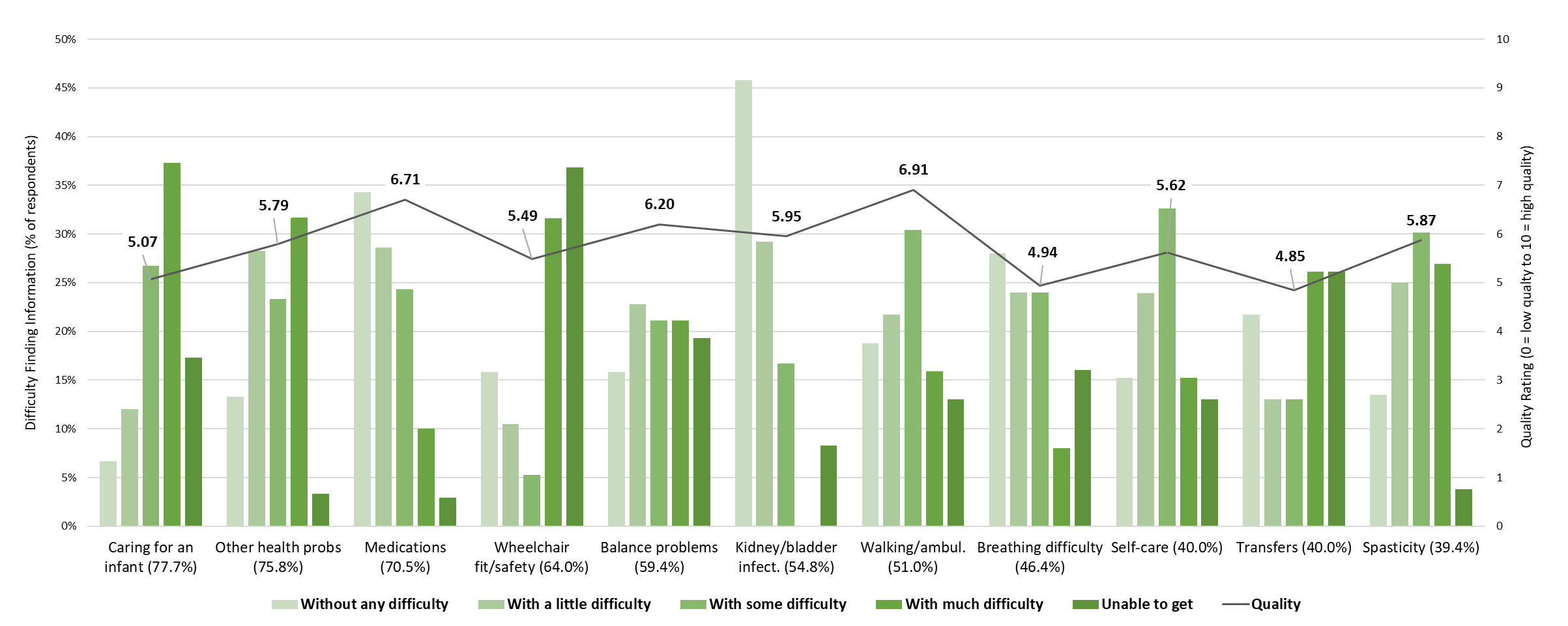
Figure S3. Difficulty Finding and Quality of Information on Pregnancy Decision-Making Topics

- % rating topic as very important is given in parentheses in the X-axis labels
- Quality ratings (right Y-axis) range from 0 = low quality to 10 = high quality

##
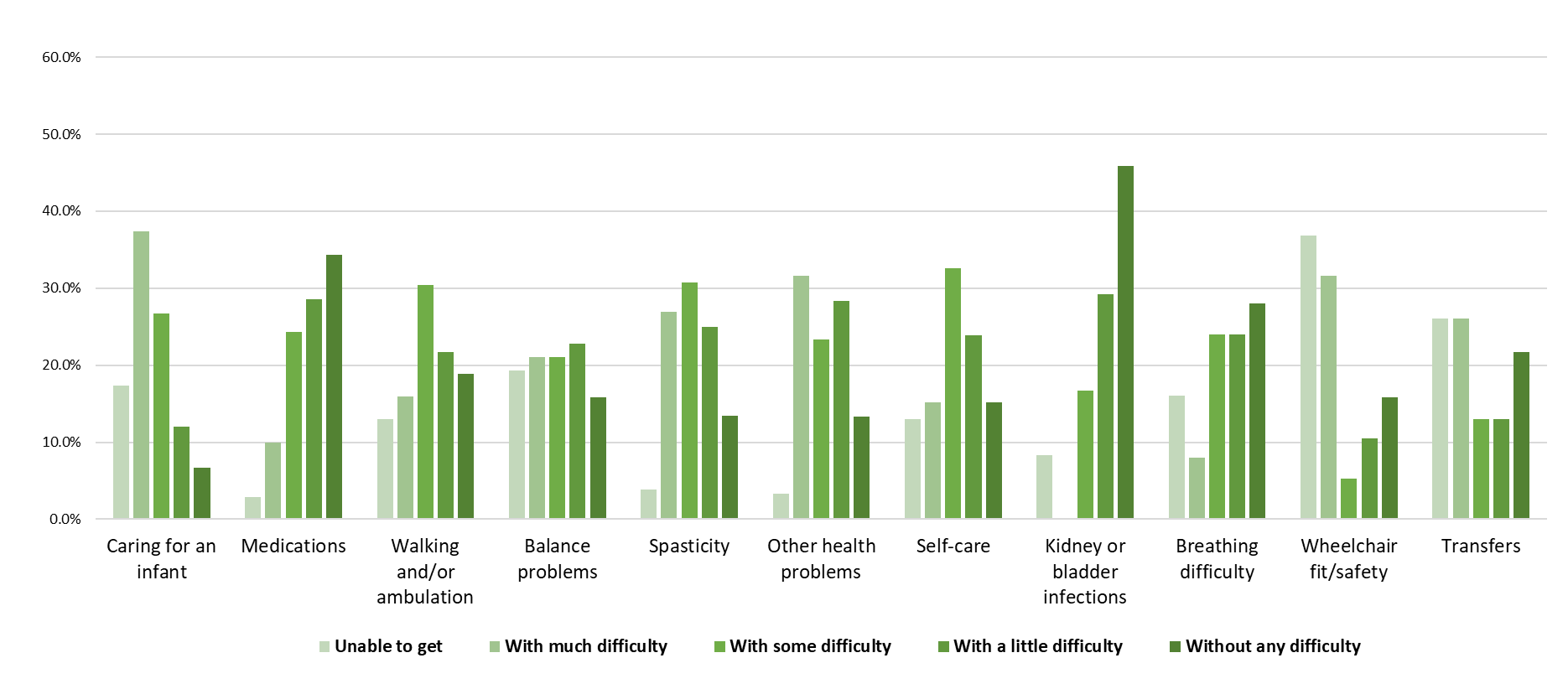
Figure S4. Ease of finding information about pregnancy among those endorsing the topic as relevant

##
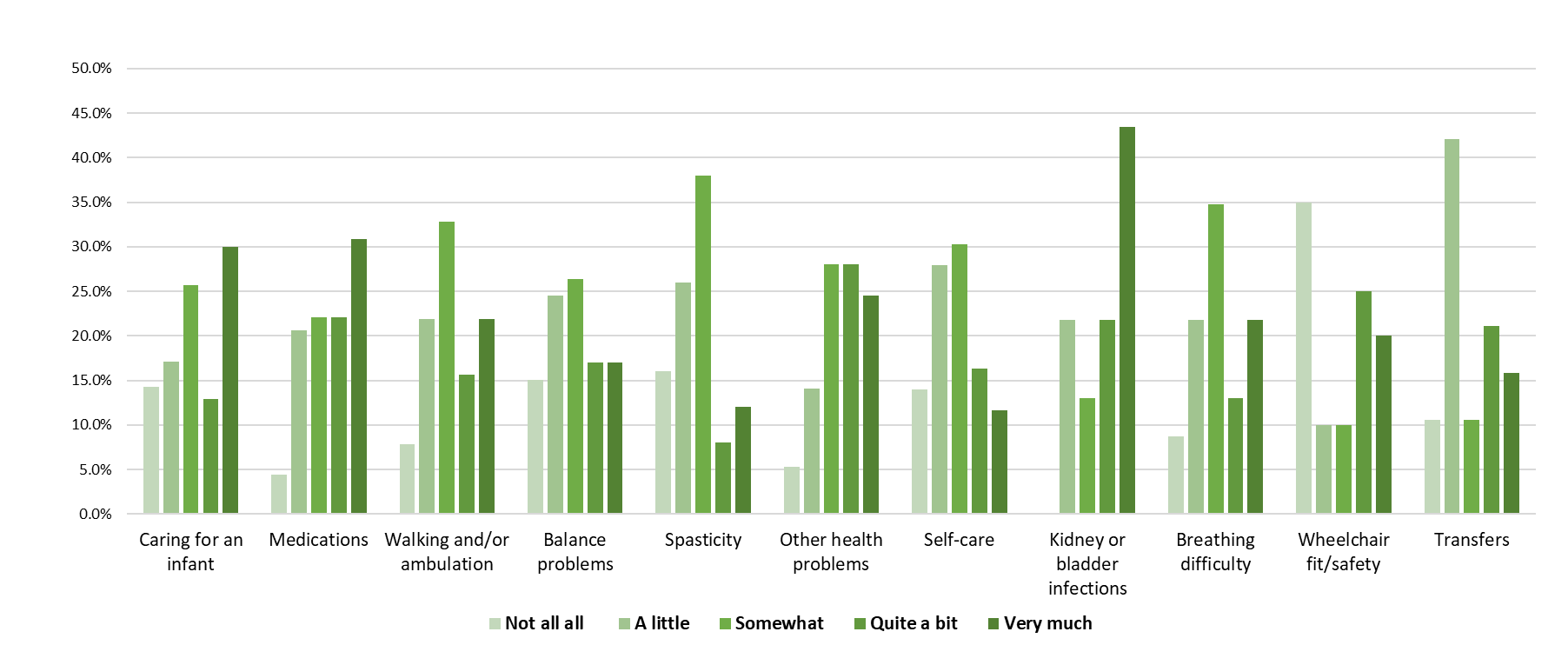
Figure S5. Helpfulness of information about pregnancy among those endorsing the topic as important

##
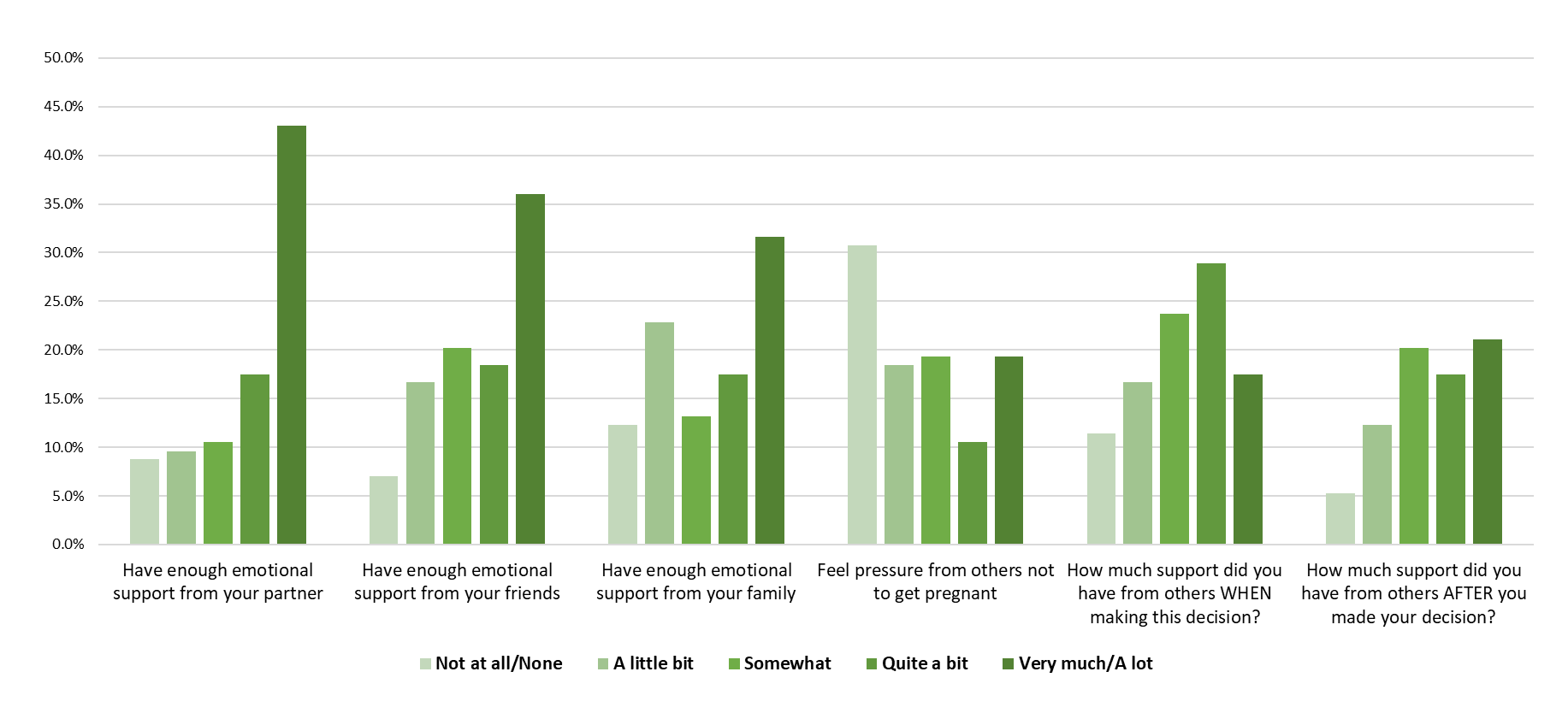
Figure S6. Support from others for decision-making about pregnancy

## Figure S7. Working with health care providers in pregnancy decision-making


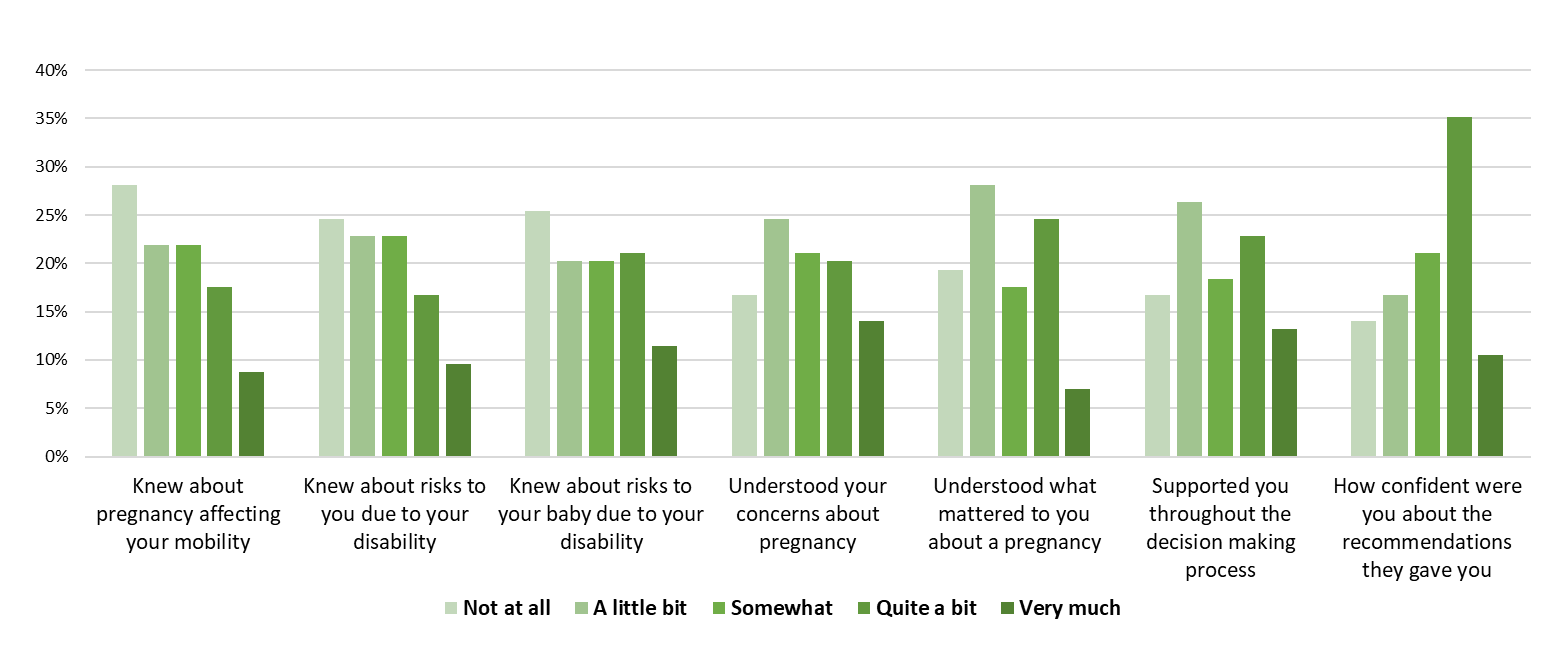

Supplement: Supplementary file 2 — Table S1: Screening questions for determining pregnancy decision‐making experience eligibility. Table S2: Themes of advice to other women with physical disabilities and healthcare providers. Figure S1: Degree of decision‐making difficulty. Figure S2: How much disability severity affected pregnancy decision‐making. Figure S3: Difficulty finding and quality of information on pregnancy decision‐making topics. Figure S4: Ease of finding information about pregnancy among those endorsing the topic as relevant. Figure S5: Helpfulness of information about pregnancy among those endorsing the topic as important. Figure S6: Support from others for decision‐making about pregnancy. Figure S7: Working with health care providers in pregnancy decision‐making. [file BJO-133-1016-s001.docx]
